# Notes and News

**Published:** 1887-06-11

**Authors:** 


### NOTES AND NEWS.

COLLECTED AND COMMUNICATED.

THUS far about £11,000, or nearly a fourth of the sum required, has been subscribed towards the building fund of the new Great Northern Hospital.

AT the sixty-sixth half-yearly election of inmates and pensioners of the Royal Hospital for Incurables, Putney, it was decided to elect fifteen additional candidates in commemoration of the Jubilee. Forty candidates were therefore elected, the usual number being twenty-five.

MISS ELEANORA LILIAN FLEURY, a student of the London School of Medicine for Women, has taken first place in the honours list, and an exhibition of £20 in the recent examination in medicine at the Royal University of Ireland.

THE Children's Jubilee Tribute, in the shape of a fund for the completion of the Ormond Street Hospital for Children, has elicited a very wide response from English children in all parts of the globe, but it is estimated that £16,000 is still required to complete the sum needed for the erection of the projected wing to this institution.

MISS BARTON, Matron to the Windsor Royal Infirmary, having been requested to fill the post of Lady Superintendent to the Royal Free Hospital, Gray's Inn Road, will be succeeded at Windsor by Miss Coke, who also holds the St. Bartholomew's certificate of training.

MISS MOLLETT, late Superintendent of Nursing at the National Hospital, Queen Square, and who was trained in the Nursing School of St. Bartholomew's Hospital, has been appointed Matron to the Chelsea Workhouse Infirmary. The Guardians are to be congratulated upon their selection, as Miss Mollett is acknowledged to be one of the most intellectual and indefatigable members of the nursing profession.

THE Jubilee celebration of the National Hospital for the Paralysed and Epileptic (Albany Memorial), Queen Square, will take place on the 7th July, when the Duchess of Albany will open the wards hitherto unoccupied, including the ward for children which bears H.R.H.'s name. This will raise the total available beds to 180. A show of roses and other flowers by Paul and Son of Waltham Cross, with additional attractions, will be provided.

WE report elsewhere the opening of the hospital campaign, which will be conducted in every part of the metropolis during the next fortnight in support of all the medical charities. At the opening meeting at Lambeth Palace an incident occurred which was as interesting as it is gratifying. The Archbishop of Canterbury, it will be seen, called attention more than once to THE HOSPITAL, and he stated after the meeting was over that "it always contained interesting and instructive matter." We were aware that our readers were drawn from all classes of the community, but it is a pleasure to find that so busy a man as the Archbishop should make a point of reading what we have to say week by week. The incident is most encouraging, and goes far to prove the paramount influence and interest exercised by the hospitals upon all thoughtful people in the present day.

AT a Tynwald Court (*i.e.*, a meeting of the Insular Legislature), held at Douglas, Isle of Man, on the 20th May last, the Douglas Town Commissioners obtained the necessary sanction to enable them to purchase, for the erection of an Infectious Hospital, an excellent site about a mile and a half from the town, at the price of £110 per acre. At the same Court a grant of £1,000 from the Insular revenue towards the purchase of the site and the building of the hospital was made, and the commissioners were authorised to borrow, for the same purposes, a sum not exceeding £2,000 on mortgage of the town rates.

HOSPITAL subjects are not entirely absent from the walls of Burlington House. Mr. F. J. Williamson sends the cast of his famous statue of Sister Dora, which is now a prominent feature of the town of Walsall. In the large gallery there is a delightful portrait, entitled "An Inexperienced Nurse," by Miss Mary Drew. The subject of this picture is Miss Enid Margery, the youthful and very pretty daughter of Mr. Frederick Treves, F.R.C.S., the well-known surgeon of the London Hospital. Miss Treves, who is habited in nurse's costume, stands by the side of a table, whereon are suggestive adjuncts of the sick-room.

THE Earl of Derby, K.G., presided at the Annual Court of Governors of the Brompton Consumptive Hospital on Wednesday. The forty-sixth annual report, read by Mr. Dobbin, the secretary, was, on the whole, satisfactory. Whilst there had been a falling off in the donations, the annual subscriptions had shown an increase. The work done by the hospital had been of the usual extensive and excellent character; nearly twenty thousand in-patients had been treated during the year 1886, and many of them had returned to their homes greatly benefited by the treatment they had received. The secretary explained that the hospital was almost entirely dependent upon voluntary contributions, and required an annual income of £24,000. This is a large sum for a special hospital; but when it is remembered that the speciality here is consumption, no one will say other than that the sum, large as it is, is spent on one of the most urgent and important objects it is possible to conceive.

THE following vacancies are announced this week, the amount of the salary being placed in each case after the name of the institution :—

**Honorary Medical Officers.**

Birmingham, Queen's Hospital. Surgeon. F.R.C.S.E.

**Resident Medical Officers.**

Bath, Royal United Hospital. Doubly-qualified.—£100.
East London Hospital for Children. Clinical Assistant. No salary.
Manchester, Ancoats Hospital. Junior Visiting Surgeon.—£60.
Royal Westminster Ophthalmic Hospital. House Surgeon. Qualified.
Toxteth Park Workhouse. Assistant. Doubly-qualified —£100.
Victoria Hospital for Children. Doubly-qualified.—£50.
Westminster Hospital. Fourth Assistant Surgeon. F.R.C.S.E.
Whitehaven Infirmary. House Surgeon. Doubly-qualified. —£120.

**Non-resident Medical Officers.**

Biggleswade Union. Three appointments.—£120, £40, £40.

**Matrons and Superintendents.**

Batley Cottage Hospital. Matron.

Birmingham Queen's Hospital. Lady Superintendent.—£75.

Leeds Hospital for Women and Children. Matron.—£40.

Margate Cottage Hospital. Matron-nurse.—£30.

**Trained Nurses.**

Birmingham Queen's Hospital. Medical Nurse.—£20.

Denbigh Infirmary. Head.—£25.

Dorchester Hospital. Head and Assistants.—£25 and £16.

Maidstone Hospital. Lady.—£25.

Mold Cottage Hospital. One.—£25.

National Hospital for the Paralysed. One.

Oldham Infirmary. Staff Nurse.

Teddington Cottage Hospital. Two.—£30.

Truro Infirmary. One.—£25.

Wrexham Infirmary. One.—£25.

**Probationers.**

Stafford Infirmary. Several.

ON Saturday, May 28th, the Prince and Princess of Wales, accompanied by their daughters, paid a visit to the Deaconesses' Institution and Hospital at Tottenham, an institution for the training of nurses and the treatment of the sick and injured. The hospital is one in which that prince of philanthropists, the late Samuel Morley, took a very deep interest. His sons, Mr. Charles and Mr. Samuel Hope Morley, have followed up their father's munificence by offering to defray the whole cost of a wing which it was proposed to build to complete the hospital. The purpose of the visit of the Prince and Princess was to open the new building, which has cost, in all, £4,500. The wing contains three wards, two for adults, containing twelve beds each, and one for children, containing thirteen beds. The Prince, in responding on behalf of the Princess to an address of welcome, read by Dr. Laseron, observed : "The Princess of Wales desires me to express her sincere thanks for the address which has just been read to her, and to express to all who take an interest in this institution the great pleasure and gratification which it gives her to take part in this day's proceedings. There can be nothing, I am sure, more noble and more praiseworthy than an institution like this, where women give up their lives to the object of philanthropy in order to heal and mitigate the sufferings of the sick. (Cheers.) An institution like the Deaconesses' is one well worthy the support of all, and I am sure the proceedings of to-day, and the opening of a fresh wing of this hospital, are a sincere gratification to the Princess, and especially in that it should be called after one whom I had the privilege of knowing, and whom you all knew, at least by name, and whose loss we must all deeply deplore—the late Samuel Morley. I feel sure no more fitting name could be given to the wing than that it should be called after him. In the name of the Princess I beg again to express the pleasure which she feels in being here to-day."

THE knowledge of an "instantaneous" remedy for lumbago would doubtless come as an inestimable boon to many poor sufferers. Professor Burggraeve, of Germany, recommends the following mixture as attended by such an effect:—R. Collodion, tincture of iodine, liquid ammonia, equal parts, M. Apply widely over the affected region with a camel's-hair brush.

DURING the past year more than the usual number of cases of sickness, caused by exposure after shipwreck, have been admitted to the wards of the Seamen's Hospital at Greenwich. Some of these cases, we learn from the sixty-sixth annual report, had been picked up in open boats at sea, and forwarded to the Seamen's Hospital by the British Consul at the first port at which the rescuing vessel touched. The total number of patients under treatment in 1886 was 2,095, and the number of out-patients relieved was 5,359. The average number of beds constantly occupied was 180, whilst the average period of stay of each patient was 31 days. The income of the Society in 1886 was £11,111 and the expenditure £11,624, so that, in spite of the fact that subscriptions to this invaluable charity are forthcoming from almost every Government in Europe excepting that of France, it is evident that further aid is required to enable it to carry on its good work in a thorough and satisfactory manner.

BEFORE the Poplar Hospital was built accidents occurring to labourers in the docks and to men engaged in the factories and great engineering works which are congregated around what we know as the "Port of London," had to be taken to the London Hospital, a distance of some two miles. In the case of serious accidents—and accidents in laborious and dangerous industries are often very serious—this tedious transport meant not infrequently death, where a life might have been saved. The Poplar Hospital, which was built in 1855, is located in the East India Dock Road, just where it is most wanted. The thirty-second annual festival in aid of this hospital was held the other evening at the Holborn Restaurant, under the presidency of Mr. Sydney Buxton. The hospital, we are happy to learn, is free from debt. Over 9,000 patients were treated last year, the numbers having nearly doubled themselves during the past five years. The committee are anxious either to establish a Convalescent Home, or to assist in supporting some similar institution already existing, where they could send some of their patients. The name of Buxton is intimately associated with the Poplar Hospital. Sir Fowell Buxton, the grandfather of Mr. Sydney Buxton, was one of the founders of the hospital, and there are now no fewer than eight living Buxtons who figure on the list of Life Governors of thi institution.

THE following sums have been recently received by the various Medical Charities, the name of the donor or the source from which they were obtained being given in each instance after the name of the Institution :—

**Donations.**

Great Northern Central Hospital. Mrs. Andrew Fernie, £400 (additional donation).
Metropolitan Convalescent Institution, Walton-on-Thames. Company of Mercers (on account of the Earl of Northampton's Charity), £105.
National Hospital for Consumption, Ventnor. Company of Mercers (on account of the Earl of Northampton's Charity), £210.
North Eastern Hospital for Children, Hackney Road. Company of Tylers and Bricklayers, £10.
North London Consumption Hospital, Hampstead. Captain Notman, £10 10*s.*
North London Hospital, Mr. D. B. Chapman, £10 10*s.* Baron de Stern, £20.
Queen Charlotte's Lying-in Hospital, Marylebone Road. Mr Simeon Lazarus, £31 10*s.*

**Entertainments.**

Great Northern Central Hospital. Proceeds of the Portman Cinderella Dances, £150.

**Legacies.**

Cancer Hospital, Fulham Road. Mrs. Maria Newen, £300.
Hospital for Consumption, Brompton. Mrs. Maria Newen, £300.
National London Temperance Hospital. Mrs. Maria Newen, £100.
Northern Hospital, Liverpool. Mrs. Anna Maria Heywood, £1,000.
National Hospital for the Paralysed and Epileptic, Bloomsbury. Mrs. Anna Maria Heywood, £500.
Royal Infirmary, Liverpool. Mrs. Anna Maria Heywood, £1,000.
West Derby Nursing Institution, Liverpool. Mrs. Anna Maria Heywood, £100.
West Derby Dispensary, Liverpool. Mrs. Anna Maria Heywood, £100.

---

ON Saturday, the 14th ult., the chairman of the Health Committee of Sunderland (Councillor Rickaby) laid the foundation-stone of the new Hospital for Infectious Diseases. The site is a very fine one, some twelve acres in extent, quite isolated from the town. Mr. Shafto is the contractor. The new hospital will cost £14,796. The site was acquired from Col. Scurfield for £5,000. The heating, ventilation, and drainage will be on the most approved system.

---

A MEETING was recently held at Barmouth, the Lord Lieutenant of Merioneth (Mr. R. D. Pryce) presiding, to consider the advisability of raising funds to establish a convalescent home in the town, for the benefit of the neighbouring counties. Dr. Lloyd stated that his experience of Barmouth, extending over a period of eighteen years, led him to the conclusion that no more suitable place for a convalescent home could be found. He had taken the trouble of observing the climatic changes which occurred there in all seasons, and he had found that during four consecutive winters the temperature ranged between forty and fifty. The death-rate had also been very low during his residence in Barmouth, as it had ranged from ten to fifteen per thousand of the population. Several other speakers expressed warm approval of the project, and a local committee was appointed to forward the scheme and raise the necessary funds. During the meeting donations to the amount of nearly one hundred and fifty pounds were promised.

THE Home Hospitals Association, Fitzroy House, Fitzroy Square, has issued its ninth annual report, which announces with satisfaction the continued efficiency and success of the institution. The number of patients admitted during the year ending December 31st, 1886, was 242, or an increase of ten over the preceding twelve months. The average cost of each patient was £13 11*s.* 9*d.*, or 11*s.* 11*d.* less than in 1885. The Committee hope, as soon as the existing debt in the hospital has been liquidated, to make a sensible reduction in the charge of some of the rooms, so that those whose income will not enable them to obtain the requisite care in their own homes may here find all that is needed by payment of a very moderate sum. It is satisfactory to learn that eighty eminent London practitioners, who have had patients in Fitzroy House during 1886, have expressed themselves to be well satisfied with its management and arrangements.

---

WE are pleased to acknowledge the receipt from Boston of the annual report of the New England Hospital for Women and Children. During the year ended September 30th, 1886, there were treated in the hospital 379 patients, a less number than in the preceding twelvemonth, owing to the wards having been closed for painting and other necessary work during February and March. No less than 5,235 out-patients also received attention, either at the hospital dispensary or in their own homes. To prevent the abuse of the charity by persons able to pay for medical attendance, each patient is obliged to sign a statement, setting forth that straitened circumstances compel him to seek medical charity. If the physician has reason to doubt the truth and honesty of the visitor, he sends the signed paper to the useful society known as the Associated Charities of Boston, for investigation. The knowledge of this possible procedure on the part of the authorities has been found to accomplish the desired result, and we think a similar plan might advantageously be set on foot in London.

---

"A PLEASANT incident," says the report of the New England Hospital, "was the reception among our internes of Dr. Amandibai Joshee, of India. This lady graduated from the Medical College of Philadelphia with honour, and applied to us for clinical instruction. As she did not dare to expose herself to the winter climate of New England, we waived our usual regulation, and received her for six months only. She was warmly welcomed by all, and began her work with great interest, but unfortunately was obliged to resign her place on account of illness. She is to return to her native country, where an important position awaits her as instructor in medicine, and as assistant in the charge of the Women's Hospital lately established by Lady Dufferin. The movement for the education of women physicians in India is of great interest and importance, and we rejoice to contribute to it by any means in our power." An expression of opinion with which THE HOSPITAL begs to express its cordial agreement.

